# BMP-2 Induced Expression of Alx3 That Is a Positive Regulator of Osteoblast Differentiation

**DOI:** 10.1371/journal.pone.0068774

**Published:** 2013-06-18

**Authors:** Takashi Matsumoto, Atsushi Yamada, Ryo Aizawa, Dai Suzuki, Masayuki Tsukasaki, Wataru Suzuki, Mutsuko Nakayama, Koutaro Maki, Matsuo Yamamoto, Kazuyoshi Baba, Ryutaro Kamijo

**Affiliations:** 1 Department of Biochemistry, School of Dentistry, Showa University, Tokyo, Japan; 2 Department of Prosthodontics, School of Dentistry, Showa University, Tokyo, Japan; 3 Department of Periodontics, School of Dentistry, Showa University, Tokyo, Japan; 4 Department of Orthodontics, School of Dentistry, Showa University, Tokyo, Japan; Sudbury Regional Hospital, Canada

## Abstract

Bone morphogenetic proteins (BMPs) regulate many aspects of skeletal development, including osteoblast and chondrocyte differentiation, cartilage and bone formation, and cranial and limb development. Among them, BMP-2, one of the most potent osteogenic signaling molecules, stimulates osteoblast differentiation, while it inhibits myogenic differentiation in C2C12 cells. To evaluate genes involved in BMP-2-induced osteoblast differentiation, we performed cDNA microarray analyses to compare BMP-2-treated and -untreated C2C12 cells. We focused on *Alx3* (aristaless-like homeobox 3) which was clearly induced during osteoblast differentiation. *Alx3*, a homeobox gene related to the *Drosophila* *aristaless* gene, has been linked to developmental functions in craniofacial structures and limb development. However, little is known about its direct relationship with bone formation. In the present study, we focused on the mechanisms of *Alx3* gene expression and function during osteoblast differentiation induced by BMP-2. In C2C12 cells, BMP-2 induced increase of *Alx3* gene expression in both time- and dose-dependent manners through the BMP receptors-mediated SMAD signaling pathway. In addition, silencing of *Alx3* by siRNA inhibited osteoblast differentiation induced by BMP-2, as showed by the expressions of alkaline phosphatase (*Alp*), *Osteocalcin*, and *Osterix*, while over-expression of *Alx3* enhanced osteoblast differentiation induced by BMP-2. These results indicate that *Alx3* expression is enhanced by BMP-2 via the BMP receptors mediated-Smad signaling and that Alx3 is a positive regulator of osteoblast differentiation induced by BMP-2.

## Introduction

Bone morphogenetic proteins (BMPs) are potent secreted signaling molecules that play a central role in bone formation. BMP-2 has been shown to induce several transcription factors that promote osteoblast differentiation, such as Runx2 and Osterix [1,2]. However, the molecular events downstream of BMP-2 signaling that result in tissue-specific gene expression and skeletal development have only been partially elucidated. To elucidate the transcriptional regulator of BMP-2-induced osteoblast differentiation, we performed cDNA microarray analyses and compared BMP-2-treated and -untreated C2C12 cells, which are known to spontaneously differentiate into myotubules, but differentiate into osteoblasts when treated with BMP-2 [3]. We focused on homeodomain containing proteins that have been known to be important mediators for the BMP-2-induced osteoblast differentiation [4,5]. Homeodomain proteins through their transactivation domain recruit other transcriptional activations or repressors that regulate expression of osteogenic genes during osteoblast differentiation [6]. We selected one of paired-like transcription factors, Alx3 (aristaless-like homeobox 3), as a gene clearly induced during osteoblast differentiation, because up-regulation of gene expression by BMP-2 is much higher than that of other homeodomain genes (microarray data have been deposited in Gene Expression Omnibus (GEO) with accession code GSE33567, http://www.ncbi.nlm.nih.gov/geo/query/acc.cgi).

*Alx3* is a homeobox gene that belongs to a group of *Aristaless*-related genes that includes *Alx4* and *Cart1* [7]. *Alx3*, *Alx4*, and *Cart1* encode highly related proteins, and their expression patterns are highly similar [7]. Alx3, originally isolated from the hamster insulinoma cell line HIT-T15 [8], participates in regulation of insulin gene expression in pancreatic β-cells through transactivation of the insulin promoter by acting on the E2A3/4 enhancer in cooperation with E47/Pan1 [9]. Alx3 is also expressed in mesenchyme of developing limbs and craniofacial regions [10–12]. The physiological roles of Alx3 have been studied using *Alx3*-null mice, a number of which died during embryogenesis [13]. *Alx3*-null mice exhibit increased failure of cranial neural tube closure and increased cell death in the craniofacial region as embryos in the absence of folic acid [13]. It has also been revealed that ALX3 is essential for normal facial development in humans and that its deficiency causes frontonasal malformation (FNM) [14]. Furthermore, Alx3 has been linked to developmental functions in craniofacial structures. However, little is known regarding its direct relationship with bone formation, especially with osteoblast differentiation.

In the present study, we focused on the mechanisms of *Alx3* gene expression and function during osteoblast differentiation induced by BMP-2.

## Results and Discussion

### BMP-2 induced *Alx3* gene expression during osteoblast differentiation in C2C12 cells

**Figure 1:**
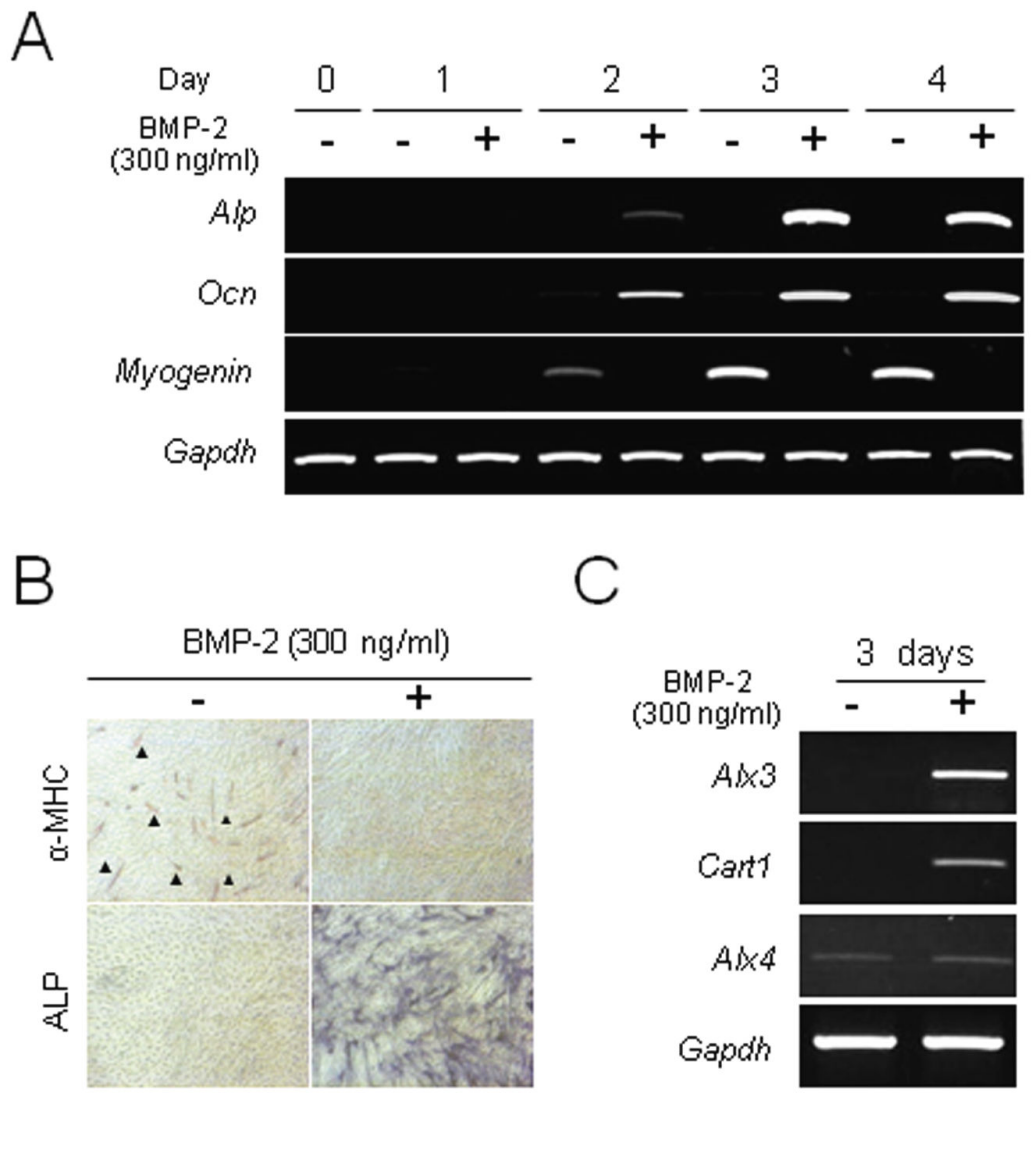
BMP-2-induced *Alx3* gene expression during osteoblast differentiation in C2C12 cells. (A) Semi-quantitative RT-PCR analyses of *Alp*, *Ocn*, and *Myogenin* gene expressions. (B) Double staining for α-MHC (*red; arrowheads*) and ALP (*blue*) as markers of differentiation for mature myotubuls and osteoblasts, respectively. (C) Semi-quantitative RT-PCR analyses of *Alx3*, *Cart1* and *Alx4* gene expressions.

C2C12 cells, one of the mouse myoblast cell lines, were used as a model of BMP-2-induced osteoblast differentiation. Cells were differentiated into osteoblasts or myotubules using low mitogen medium with or without BMP-2 [15]. First, we checked the gene expressions of *Alp*, *Osteocalcin*, and *Myogenin* after treating C2C12 cells with or without BMP-2 (Figure 1A). Time course analysis revealed that C2C12 cells differentiated into myotubules without BMP-2, as the expression of *Myogenin* was increased in a time-dependent manner. On the other hand, the cells differentiated into osteoblasts with BMP-2, as the expressions of *Alp* and *Osteocalcin* were increased in a time-dependent manner. Furthermore, analyses of ALP activity and α-MHC immunohistochemistry also revealed that C2C12 cells were differentiated into osteoblasts or myotubules with or without BMP-2 treatment, respectively (Figure 1B). These results are consistent with the previous report [15]. To elucidate the transcriptional regulator for BMP-2-induced osteoblast differentiation, we performed cDNA microarray analysis to compare between BMP-2-treated and -untreated C2C12 cells (GEO accession code GSE33567). Among them, we selected *Alx3*, a homeobox gene that belongs to a group of *Aristaless*-related genes, as it was clearly induced during osteoblast differentiation (Figure 1C). Previous studies have indicated that homeobox genes including *Dlx3*, *Dlx5*, and *Msx2* are important for regulation of osteoblast differentiation, while some are up-regulated in C2C12 cells during BMP-2-induced osteoblast differentiation (Table S1) [16,17]. Furthermore, the expressions of *Cart1*and *Alx4*, which belong to the same protein family as Alx3, were examined during BMP-2-induced osteoblast differentiation. An increased expression of *Cart1*, but not of Alx4, was found, which was similar to that of Alx3 during BMP-2-induced osteoblast differentiation (Figure 1C).

### BMP-2 induced *Alx3* gene expression in time- and dose- dependent manners

**Figure 2:**
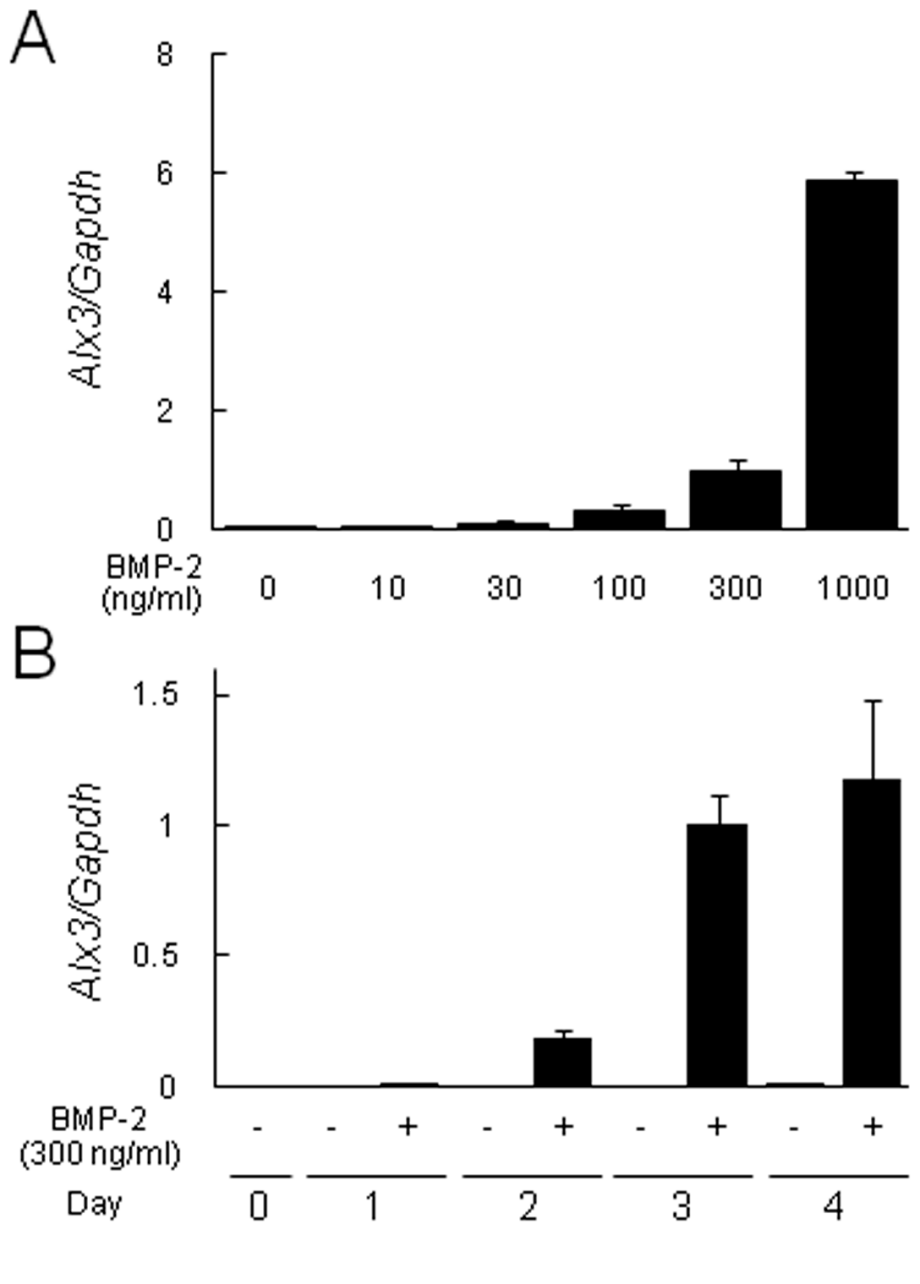
BMP-2 induced *Alx3* expression in time- and dose- dependent manners through the Smad signaling pathway. (A) Dose effects of BMP-2 on *Alx3* expression. C2C12 cells were treated with 10, 30, 100, 300, or 1000 ng/ml BMP-2 for 3 days. (B) Time course analysis of BMP-2 effects on *Alx3* expression. C2C12 cells were treated with or without 300 ng/ml of BMP-2 for 1, 2, 3, or 4 days.

The regulation of *Alx3* gene expression by BMP-2 was examined in more detail. When C2C12 cells were treated with various concentrations of BMP-2 for 3 days, *Alx3* gene expression was enhanced in the presence of 10 ng/ml of BMP-2 and then increased in a dose-dependent manner (Figure 2A). Next, time course analysis of BMP-2 was conducted with a fixed concentration of 300 ng/ml at various time points between 1 and 4 days. Increased Alx3 expression was detected 1 day after addition of BMP-2, which gradually increased up to 4 days (Figure 2B). These findings demonstrated that the BMP-2-induced increase of *Alx3* gene expression occurred in both time- and dose-dependent manners.

### BMP-2 induced *Alx3* gene expression through BMP receptors mediated-SMAD signaling pathway

**Figure 3:**
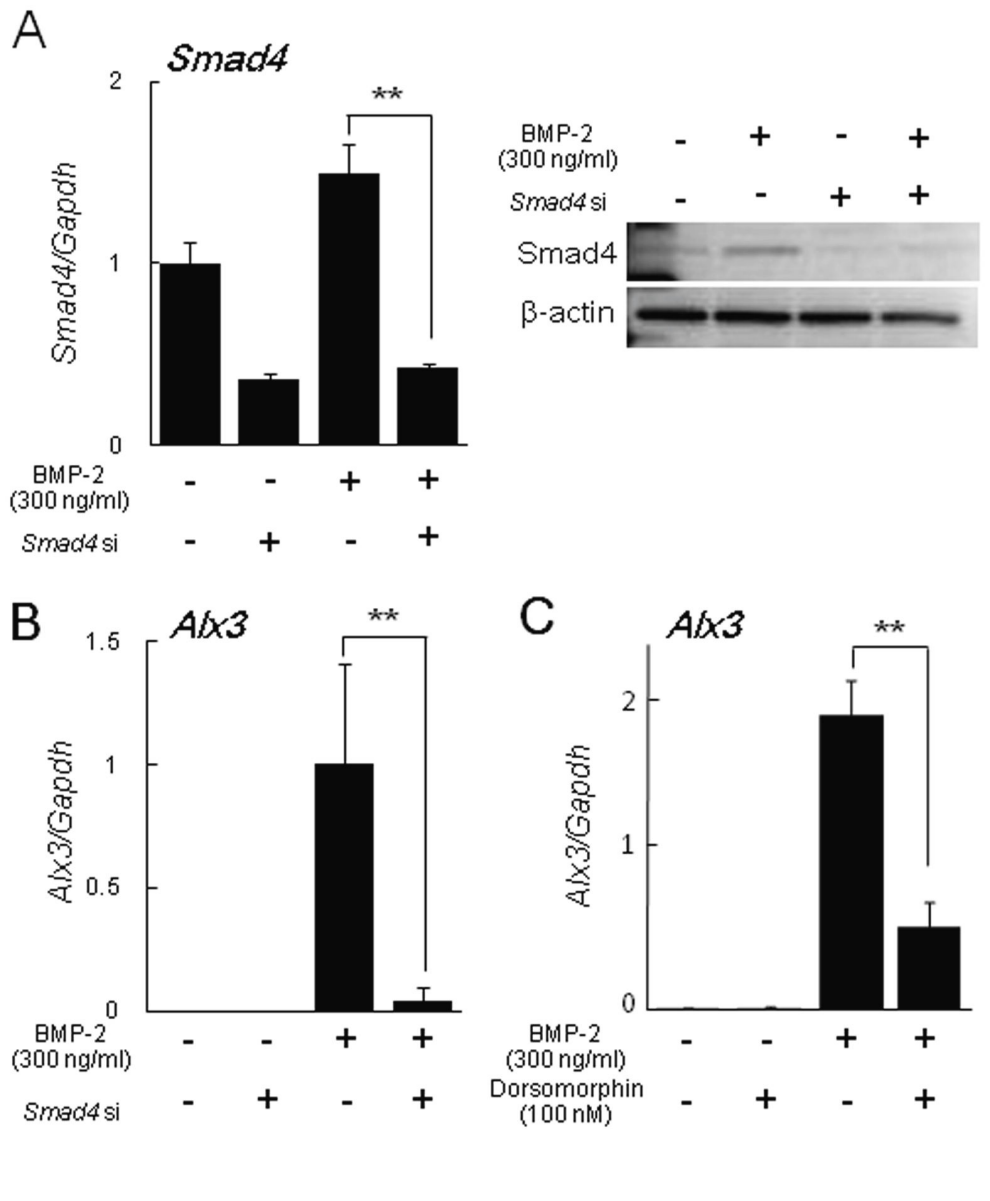
BMP-2 induced *Alx3* expression through the SMAD signaling pathway. C2C12 cells were pretreated with *Smad4* siRNA and 100 nM of Dorsomorphin, followed by treatment with or without BMP-2 for 3 days. (A) The expression of *Smad4* was examined by real-time PCR and Western blotting. (B, C) The expression of *Alx3* was examined by real-time PCR. ** *p* < 0.01 by Student’s *t* test.

Smad4, a key intracellular mediator in BMP signaling, forms transcriptional activator complexes with BMP-restricted SMAD proteins [18]. To investigate whether induction of *Alx3* gene expression by BMP-2 is dependent on the SMAD signaling pathway, we performed ablation of Smad4 in C2C12 cells using an siRNA knockdown system. First, we checked mRNA and protein levels of Smad4, when treated with *Smad4* siRNA. Treatment with Smad4 siRNA caused significant decrease levels of *Smad4* mRNA and protein in BMP-2-treated C2C12 cells (Figure 3A). Ablation of Smad4 in C2C12 cells abrogated *Alx3* gene expression induced by BMP-2 (Figure 3B). Furthermore, we used Dorsomorphin which blocks BMP induced Smad1/5/8 phosphorylation in a dose dependent manner, while having no effect on TGF-β or Activin induced Smad2/3 activation [19]. Treatment with Dorsomorphin abolished *Alx3* gene expression induced by BMP-2 (Figure 3C). These findings suggest that induction of *Alx3* gene expression by BMP-2 is dependent on the BMP receptor mediated-SMAD signaling pathway.

### Effects of knockdown and over-expression of Alx3 on osteoblast differentiation induced by BMP-2

**Figure 4:**
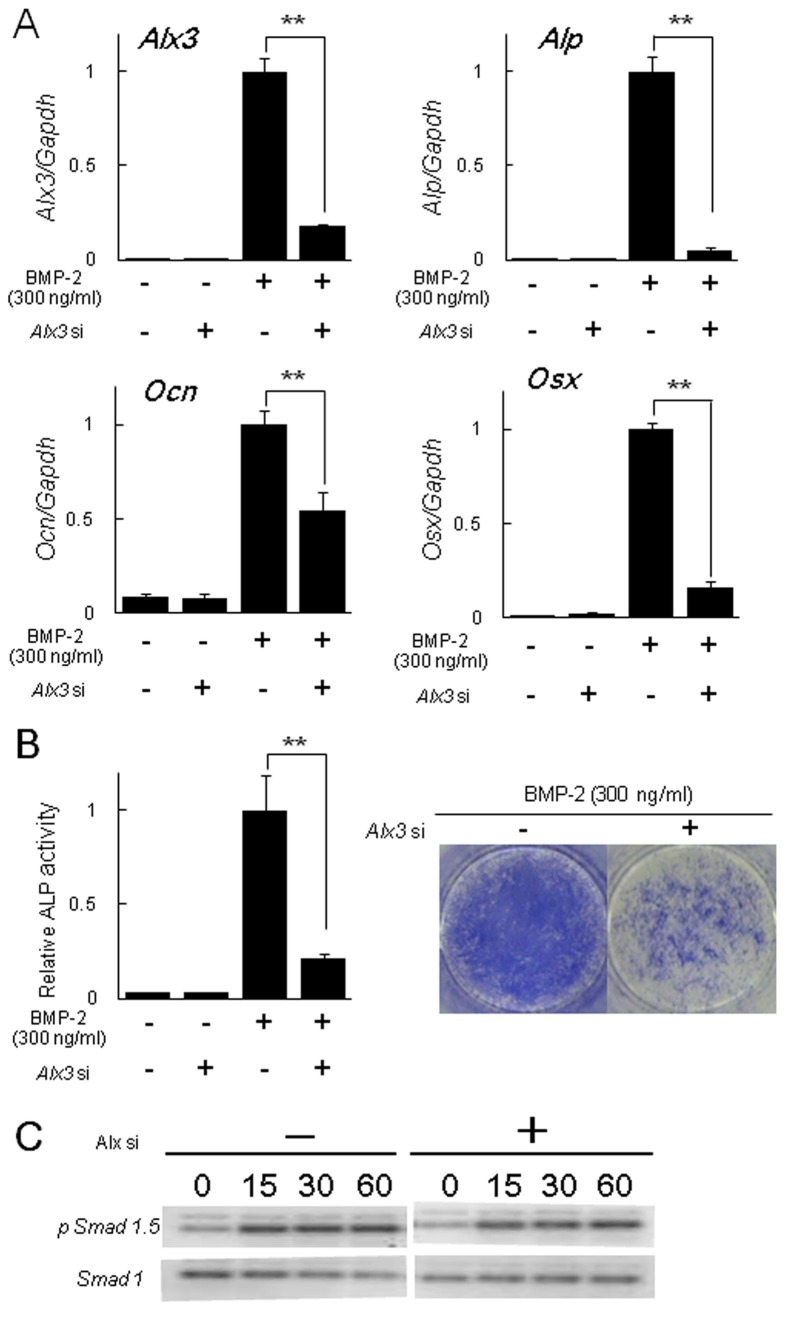
Effect of *Alx3* siRNA knockdown on BMP-2-induced osteoblast differentiation. (A) C2C12 cells were pretreated with *Alx3* siRNA, followed by treatment with or without BMP-2 for 3 days. The expressions of *Alx3*, *Alp*, *Ocn* (*Osteocalcin*), and *Osx* (*Osterix*) were quantified by real-time PCR. (B) Measurement of ALP activity and ALP staining. ** *p* < 0.01 by Student’s *t* test. (C) Effect of Alx3 siRNA knockdown on BMP-2-induced phosphorylation of Smad1/5. C2C12 cells were pretreated with *Alx3* siRNA, followed by treatment with BMP-2 for 15, 30, and 60 minutes.

**Figure 5:**
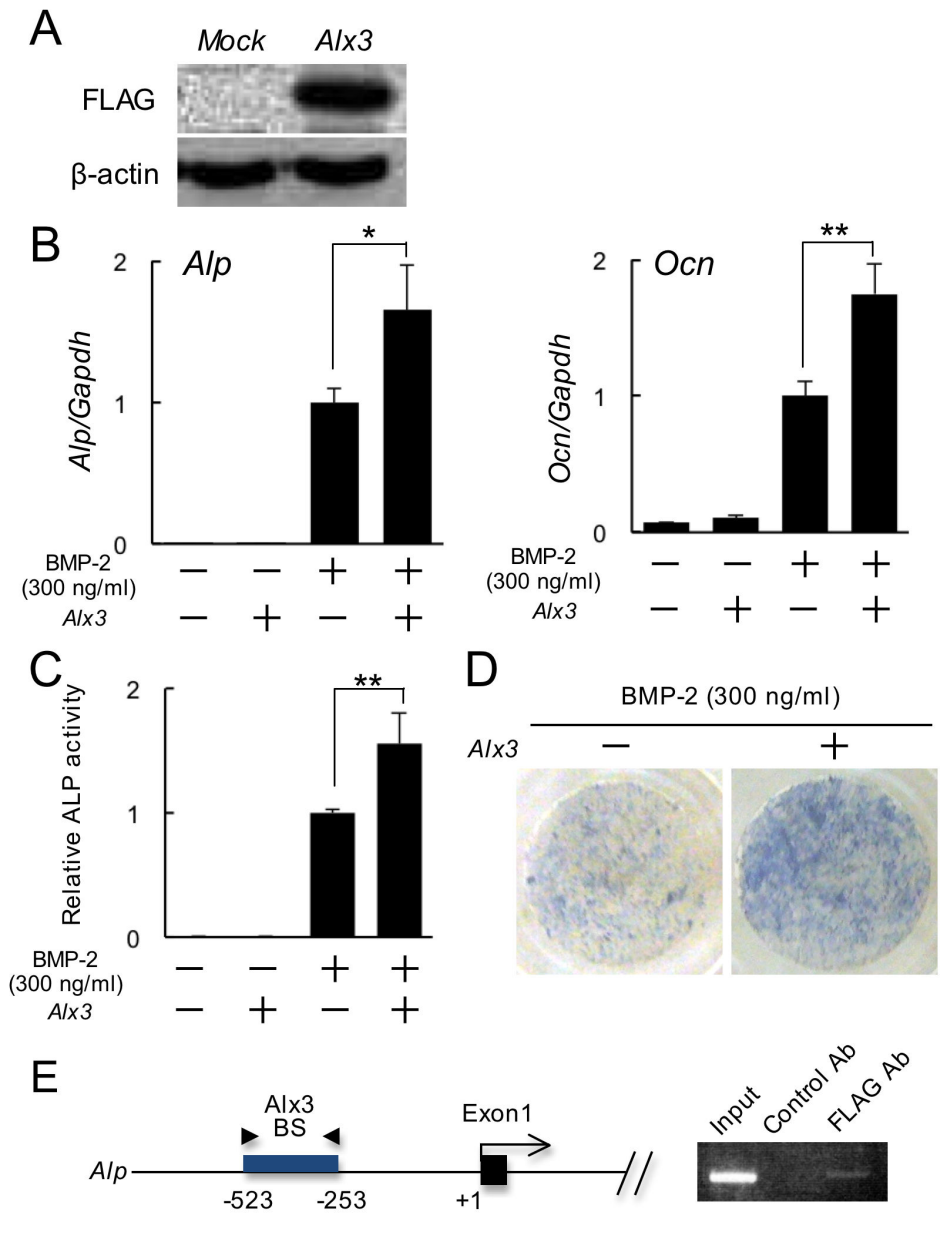
Effect of Alx3 over-expression on BMP-2-induced osteoblast differentiation. (A) Western blot analysis of Alx3 was performed using C2C12 cells transfected with empty (*Mock*) or *Alx3-FLAG* expression (*Alp*) vectors. Equal protein loading was documented by blotting for β-actin. (B) The expressions of *Alp* and *Ocn* were quantified by real-time PCR. (C, D) Measurement of ALP activity and ALP staining. ** *p* < 0.01, * *p* < 0.05 by Student’s *t* test. (E) Schematic diagram of upstream region of mouse *Alp* gene showing locations of putative Alx3-binding sites tested in ChIP analyses. Arrowheads indicate the positions of the primers used for ChIP analysis. ChIP analyses were performed using DNA fragments immunoprecipitated with a FLAG antibody or isotype-specific control antibody. Immunoprecipitates were PCR amplified with primers flanking the putative Alx3-binding region. Ab, antibody; BS, binding site.

To determine whether Alx3 is involved in BMP-2-induced osteoblast differentiation, we knocked down *Alx3* gene expression induced by BMP-2 using *Alx3* siRNA and investigated osteoblast marker gene expression in C2C12 cells. The expressions of *Alp*, *Osteocalcin*, and *Osterix* induced by BMP-2 were remarkably decreased (Figure 4A). Analyses of ALP activity also revealed that BMP-2-induced osteoblast differentiation was inhibited by *Alx3* siRNA treatment (Figure 4B). Here, we should consider whether down-regulation of Alx3 affects the BMP-mediated early intracellular signaling or BMP-2-induced osteogenic responses. We checked the phosphorylation of Smad1/5, canonical BMP-signaling molecules, induced by BMP-2 in Alx3 siRNA-treated C2C12 cells. The phosphorylation levels of Smad1/5 were not different between Alx3 siRNA treated or non-treated C2C12 cells induced by BMP-2 (Figure 4C). These results suggest that down-regulation of *Alx3* gene expression inhibited BMP-2 induced osteogenic responses. Furthermore, we over-expressed Alx3 in C2C12 cells using a FLAG tagged *Alx3* expression vector (Figure 5A), which caused increases in the expressions of *Alp* and *Osteocalcin* (Figure 5B). Also, analysis of ALP activity revealed that BMP-2-induced osteoblast differentiation was increased in C2C12 cells with Alx3 over-expression (Figure 5C, D). These findings suggest that Alx3 is a positive regulator of BMP-2 induced osteoblast differentiation.

To identify the actual targets of Alx3 functions, we performed chromatin immunoprecipitation combined with DNA microarray analysis (ChIP-on-chip). Selected genes identified by ChIP-on-chip analysis are listed in Table S2, along with the genomic location of the Alx3 binding site within 10 kbp upstream of the transcriptional starting sites and enrichment log (base e) ratio (GEO accession code GSE44822). Among them, *Alp*, whose promoter contains homeodomain-binding cis-regulatory elements with a TAAT motif (-431/-434 bp of the *Alp* promoter) [20], was validated by ChIP-PCR findings (Figure 5E). These results suggest that *Alp* is one of the target genes directly regulated by Alx3.

### Possible mechanisms of BMP-2 induction of *Alx3* expression and functions of Alx3 in BMP-2-induced osteoblast differentiation

Our findings demonstrated that the expression of Alx3, a critical regulator of osteoblast differentiation, in C2C12 cells is enhanced by treatment with BMP-2. *Alx3* expression was downregulated by treatment with Smad4 siRNA, suggesting that Alx3 induction by BMP-2 is directly or indirectly regulated by Smad signaling. Actually, within -10 Kbp from the transcription start site of the *Alx3* gene, 12 putative Smad responsible elements were detected by use of a transcription factor search program (TRANSFAC). Recently, Garcia-Sanz P. *et al.* demonstrated that the Alx3 promoter is regulated by the basic helix-loop-helix (bHLH) transcription factors Twist1 and upstream stimulatory factor 1, 2 (USF1, 2) in mesenchymal cells [21]. Although the mechanism by which BMP-2 promotes SMAD-dependent expression of *Alx3* remains to be determined, SMAD signaling induces *Alx3* expression, which may act as a regulator of the positive feedback loop to BMPs.

We also found that ablation of *Alx3* inhibited BMP-2-induced osteoblast differentiation, while over-expression of Alx3 promoted that in C2C12 cells. Mirasierra *et al.* demonstrated that Alx3 participates in regulation of insulin gene expression in pancreatic β-cells by physiologically interacting with E47/Pan1 [9]. E47 is one of the alternative spliced products of E2A and belongs to the bHLH family of transcriptional regulatory proteins [22]. To investigate the effect of E47 on Alx3-enhanced osteoblast differentiation induced by BMP-2, we performed co-transfection with Alx3 and E47 expression vectors in BMP-2-treated C2C12 cells. However, that co-transfection did not significantly modify the expressions of *Alp* and *Ocn*, or ALP activity (Figure S1). Furthermore, we examined the effect of Cart1, which is known to play a crucial role in the developmental lineage of bone and cartilage, and also regulates transcriptional activity together with Alx3 in regard to osteoblast differentiation enhanced by Alx3 [23]. Co-transfection with Alx3 and Cart1 expression vectors did not significantly modify the expressions of *Alp* and *Ocn*, or ALP activity (Figure S1). Further studies are required to determine the partners of Alx3 for enhancement of osteoblast differentiation.

We concluded that Alx3 expression is enhanced by BMP-2 via a mechanism that is dependent on SMAD signaling. In addition, Alx3 is also involved in osteoblast differentiation of C2C12 cells induced by BMP-2.

## Materials and Methods

### Reagent

Dorsomorphin was purchased from SIGMA.

### Cell culture

Murine C2C12 skeletal myoblasts were purchased from RIKEN BioResource Center (C2C12 #RCB0987, Tsukuba, Japan). C2C12 cells were grown in Dulbecco’s modified Eagle’s medium (DMEM; Wako Pure Chemical Industries) supplemented with 15% fetal bovine serum (FBS) (growth medium). To examine the effects of BMP-2 (provided by Astellas Pharmaceuticals) on myoblast and osteoblast differentiation of C2C12 cells, the growth medium was replaced with DMEM supplemented with 2.5% FBS (differentiation medium).

### RT-PCR

Total RNA was extracted from C2C12 cells with TRIzol reagent (Life Technologies). First strand cDNA was synthesized from total RNA using a SuperScript First Strand Synthesis System with SuperScript III reverse transcriptase (Life Technologies). The generated cDNA was used as a template for semi-quantitative and real-time PCR assays with an ABI StepOne^TM^ Real Time PCR system (Life Technologies). The following primers were used for semi-quantitative PCR: *Alp*, GATCATTCCCACGTTTTCAC and TGCGGGCTTGTGGGACCTGC; *Osteocalcin*, CAAGTCCCACACAGCAGCTT and AAAGCCGAGCTGCCAGAGTT; *Myogenin*, GGGCCCCTGGAAGAAAAG and AGGAGGCGCTGTGGGAGT; *Cart1*, CCCTTGTAAAGTGATACCTG and CAAGATACGTTTGTATGCGA; *Alx3*, CTCCATGCATGTCCCCATAC and GCCCTAAGAACCACAGAGAG; *Alx4*, CCTGGATTGGCAACAATGGG and AGGGACAAGAGCCTACCATC; and *Gapdh*, ACCACAGTCCATGCCATCAC and TCCACCACCCTGTTGCTGTA. The following primers were used for SYBR green real-time PCR: *Alp*, GGGACTGGTACTCGGATAACGA and CTGATATGCGATGTCCTTGCA; *Osteocalcin*, CCTCTCGACCCGACTGCAGATC and AGCTGCAAGCTCTCTGTAACCATGAC; *Osterix*, CGGCCCTGAGTCTGACAAA and GCCGGAGTCTGTTCACTACCTT; and *Smad4*, GCTTGGGTCAACTCTCCAATG and TGTGCAACCTCGCTCTCTCA. The following assay IDs were used for TaqMan real-time PCR: *Alx3*, Mn00431779m1 and *Gapdh*, Mn03302249g1.

### Alkaline phosphatase activity

Alkaline phosphatase (ALP) activity was determined as a marker of osteoblast differentiation. After removing culture medium, cell layers were washed with PBS, then sonicated in 50 mM Tris-HCl (pH 7.5) containing 0.1% TritonX-100. ALP activity in the lysates was determined following incubation with the substrate, *p*-nitrophenylphosphate, in buffer (pH 10) containing 0.1M 2-amino-2-methyl-1-propanol and 2 mM MgCl_2_. The reaction was terminated by adding NaOH and values were determined at 405 nm [24].

### Histochemical analysis

For a histochemical examination of alkaline phosphatase activity, cells were fixed with 10% formaldehyde. After washing with PBS, the cells were incubated with ALP activity solution containing 0.1 mg/ml of naphthol AS-MX phosphate, 0.5% *N*,*N*-dimethylformamide, 2 mM MgCl_2_, and 0.6 mg/ml of fast blue BB salt in 0.1 mM Tris-HCl (pH 8.5). To perform immunohistochemical analysis of the expression of α-myosin heavy chain (αMHC), cells were fixed with 10% formaldehyde. They were then washed with PBS and treated with an acetone/ethanol mixture (50:50, v/v), and incubated with mouse anti-MHC monoclonal antibody (MF-20, Developmental Studies Hybridoma Bank). The reaction products were visualized using an AEC substrate kit (#415184, NICHIREI Biosciences) [24].

### Short interfering RNA (siRNA) knockdown of gene expression

C2C12 cells (50% confluence) were seeded and transfected with a 10-pmol/cm^2^ culture surface area of the siRNA pool (Stealth siRNA, Life Technologies) using Lipofectamine RNAi MAX reagent (Life Technologies) in OPTI-MEM (Life Technologies). The following IDs were used for siRNAs; Smad4, MSS206435 and Alx3, MSS201972.

### Expression vectors of Alx3, Cart1, and E47

cDNA encoding mouse Alx3 was amplified using PCR with primers 5’-AGCTCCGGCGGCCTGTGA-3’ and 5’-GTAACCCATGACGTGGTCCA-3’, and subcloned into a pCRII-TOPO TA cloning vector. Construction of an expression plasmid containing cDNA encoding mouse Alx3 was performed, then cloned into the EcoRI site of a p3X-FLAG-CMV-14 expression vector. cDNA encoding mouse Cart1 was amplified using PCR with the primers 5’-GAGCTCGCGGACAGCCTTTC-3’ and 5’-GTATGTTTCATGGCCCATGA-3’, then subcloned into a pCRII-TOPO TA cloning vector. Construction of an expression plasmid containing cDNA encoding mouse Cart1 was performed, then cloned into the EcoRI site of a p3X-FLAG-CMV-14 expression vector. The E47 expression vector was a kind gift from Dr. Mario Vallejo [9,25].

### Western blotting

Total proteins were resolved by SDS-PAGE and blotted onto PDEF membranes (Milipore). Smad1, Smad4, Smad5, and phosphor-Smad1/5 immunoreactivities were detected with a rabbit polyclonal primary antibody (1:1000 dilution) (Cell Signaling), followed by incubation with an anti-rabbit peroxidase-conjugated secondary antibody (1:2000 dilution) (GE Healthcare). FLAG immunoreactivity was detected with a mouse monoclonal primary antibody (1:1000 dilution) (SIGMA), followed by incubation with an anti-mouse peroxidase-conjugated secondary antibody (1:2000 dilution) (GE Healthcare). β-actin immunoreactivity was detected with a rabbit polyclonal primary antibody (1:5000 dilution) (SIGMA), followed by incubation with an anti-rabbit peroxidase-conjugated secondary antibody (1:10000 dilution) (GE Healthcare). Immunoreactive bands were visualized using an enhanced chemiluminescence detection system (ECL Plus, GE Healthcare) [26].

### Chromatin immunoprecipitation assays

Chromatin immunoprecipitation (ChIP) assays were performed using an EpiQuik TM Chromatin Immunoprecipitation Kit (Epigentek Group Inc., Brooklyn, NY USA) based on the protocol provided by the supplier. Briefly, 10^6^ C2C12 cells were treated with BMP-2 and chemically cross-linked by addition of a one-tenth volume of fresh 11% formaldehyde solution for 15 minutes at room temperature. Cells were then rinsed twice with 1x PBS, resuspended in CP3 lysis buffer containing a protease inhibitor cocktail (PIC), and sonicated until crosslinked chromatin was sheared to an average DNA fragment molecular length of 200-1000 bp. One percent of the sonicated lysate was used to quantify the total amount of DNA present in different samples before immunoprecipitation (input).

The sonicated samples were immunoprecipitated with the FLAG antibody (Sigma) using an EpiQuik TM Chromatin Immunoprecipitation Kit (Epigentek). Crosslinking between DNA and proteins was reversed by heating the samples at 65 ºC for 15 minutes, followed by proteinase K digestion at 65 ºC for 1.5 hours. After cleaning on spin columns, DNA was eluted in 10 mM Tris-EDTA buffer. We assayed for the presence of a putative target site in the immune complexes by PCR using primers amplifying the following genomic regions: mouse *Alp* promoter Alx3 binding site (Alx3 BS), 5’-GTCTGTGAACCCACCTGGCT-3’; -253/-272 bp, and 5’-CCACTGCCTGGGAAGTGGTC-3’; -523/-504 bp, based on the mouse *Alp* gene transcription start site as +1. The positions of the PCR fragments corresponded to those in the Ensemble Genome Browser.

### ChIP-on-chip microarray analysis

Immunoprecipitated and input DNA were amplified using a whole genome amplification kit (GenomePlex® Complete Whole Genome Amplification Kit, Sigma, USA), as recommended by the manufacturer. Amplified samples were purified using a QIAQuick PCR clean-up kit (Qiagen). Labeling reactions were performed with 4 µg of purified amplified DNA and a Bioprime labeling kit (Invitrogen), according to the manufacturer’s instructions, in a volume of 50 µl with a modified dNTP pool containing 120 µM each of dATP, dGTP, and dCTP; 60 µM dTTP; and 60 µM Cy5-dUTP (for immunoprecipitated DNA) or Cy3-dUTP (for input DNA). Labeled targets were subsequently purified using a QIAQuick PCR clean-up kit (Qiagen). Next, dye-labeled DNA samples were quantified using an ND-1000 spectrophotometer (NanoDrop Technologies, Inc., Wilmington, DE). After checking labeling efficiency, each 2.5-5 µg of the Cy 3- and Cy 5-labeled DNA targets were mixed and then resuspended with 2x hybridization buffer, Cot-1 DNA, and Agilent 10x blocking agent, along with de-ionized formamide. Before hybridization to the array, the 260-µl hybridization mixtures were denatured at 95 ºC for 3 minutes and incubated at 37 ºC for 30 minutes, then centrifuged at 17 900 x g for 1 minute and directly pipetted onto a Mouse Promoter 1x1 M microarray (Agilent Technologies, USA). The arrays were hybridized at 65 ºC for 40 hours using an Agilent Hybridization oven (Agilent Technologies, USA). The hybridized microarray was washed according to the manufacturer’s washing protocol (Agilent Technologies, USA).

### Data acquisition and analysis

Acquired hybridization images were analyzed using an Agilent DNA microarray Scanner (Agilent Technologies, USA) and data quantification was performed using Agilent Feature Extraction software (Agilent Technologies, USA). Preprocessing of raw data and normalization steps were performed using Agilent Genomic Workbench software, according to the manufacturer’s instructions (Agilent Technologies, USA). Background corrected intensity data were normalized with blank subtraction followed by intra-array LOWESS normalization, while the Whitehead Error Model v 1.0 was used to calculate confidence values for each spot on the array. The Whitehead per-array neighborhood model v 1.0 was used to identify bound regions. Criteria for identification of a positive probe were as follows(1). The P-value for the probe sets (probe and its 2 immediate neighbors) was less than 0.001, and (2) 1 of 3 probes in the probe set had a single probe P-value less than 0.005, or the center probe in the set had a single probe P-value less than 0.001 and 1 of the neighboring probes had a single P-value less than 0.1. Raw data are available online at Gene Expression Omnibus (GEO accession number GSE44822).

### Accession numbers

Microarray data have been deposited in Gene Expression Omnibus (GEO) under accession codes GSE33567 [3] and GSE44822.

## Supporting Information

Figure S1 Neither E47 nor Cart1 enhanced BMP-2-induced osteoblast differentiation by Alx3. (A) Alx3, E47, and Cart1 were overexpressed in C2C12 cells, then the cells were treated with or without BMP-2 for 3 days. Expressions of *Alp* and *Ocn* (*Osteocalcin*) were quantified using real-time PCR. (B) Measurement of ALP activity. **p < 0.01, NS: difference not significant, as shown by Student’s *t* test.
(TIF)

Table S1 Expressions of Homeobox genes in BMP-2 treated and untreated C2C12 cells. (XLSX)

Table S2 Alx3 target genes identified by ChIP-on-chip analysis. The location was relative to the annotated transcriptional starting site.
(XLSX)
