# Supplementary figures and images for: BMP-2 Induced Expression of Alx3 That Is a Positive Regulator of Osteoblast Differentiation
Source: PLoS One. 2013 Jun 18;8(6):e68774. doi: 10.1371/journal.pone.0068774 (PMC3689002; doi:10.1371/journal.pone.0068774)

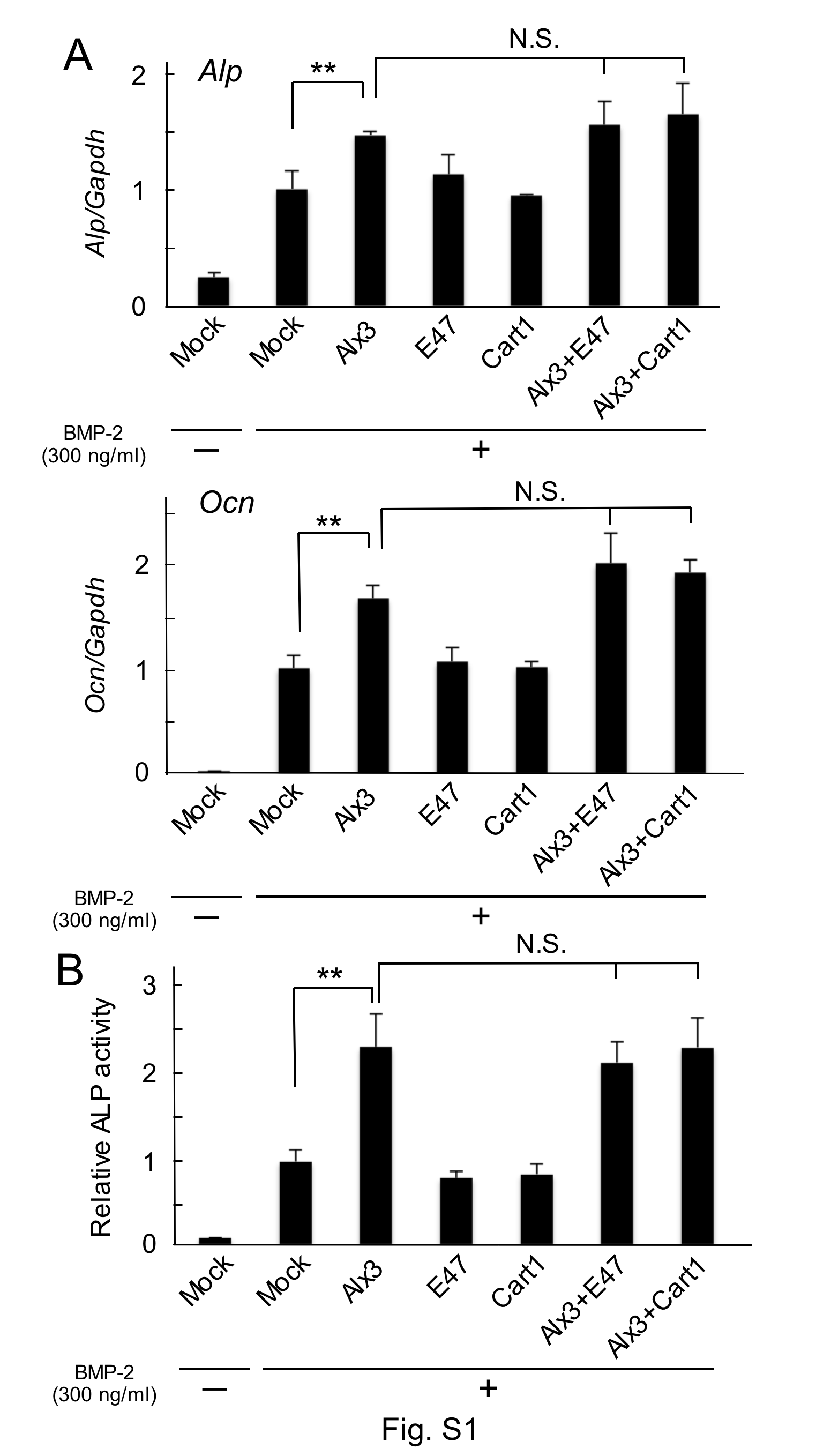

Supplement: Figure S1 — (A) Alx3, E47, and Cart1 were overexpressed in C2C12 cells, then the cells were treated with or without BMP-2 for 3 days. Expressions of Alp and Ocn (Osteocalcin) were quantified using real-time PCR. (B) Measurement of ALP activity. **p < 0.01, NS: difference not significant, as shown by Student’s t test. (TIF) [file pone.0068774.s001.tif]
